# The combination of bFGF and CHIR99021 maintains stable self-renewal of mouse adult retinal progenitor cells

**DOI:** 10.1186/s13287-018-1091-y

**Published:** 2018-12-13

**Authors:** Caixia Jin, Qingjian Ou, Zongyi Li, Juan Wang, Jieping Zhang, Haibin Tian, Jing-Ying Xu, Furong Gao, Lixia Lu, Guo-Tong Xu

**Affiliations:** 1 Department of Ophthalmology of Shanghai Tenth People’s Hospital, and Tongji Eye Institute, Tongji University School of Medicine, Shanghai, 200072 China; 2 Department of Regenerative Medicine and Stem Cell Research Center, Tongji University School of Medicine, Shanghai, 200092 China; 3 Department of Pharmacology, Tongji University School of Medicine, Shanghai, 200092 China; 4 Shandong Provincial Key Laboratory of Ophthalmology, Shandong Eye Institute, Shandong Academy of Medical Sciences, Qingdao, 266071 China; 5 Collaborative Innovation Center for Brain Science, Tongji University, Shanghai, 200092 China

**Keywords:** Retina, Progenitor cells, Wnt pathway, Cellular therapy, Transplantation

## Abstract

**Background:**

Millions of people are affected with retinal diseases that eventually cause blindness, and retinal progenitor cell (RPC) transplantation is a promising therapeutic avenue. However, RPC expansion and the underlying regulation mechanisms remain elusive.

**Methods:**

Adult mouse neural RPCs (mNRPCs) were isolated and amplified with the combination of basic fibroblast growth factor (bFGF) and glycogen synthase kinase 3 (GSK3) inhibitor CHIR99021. The progenitor characteristics were evaluated with RT-PCR, immunocytochemistry (ICC), western blot, flow cytometry, and transcriptome analysis prior to transplantation. By treating cells with or without bFGF and CHIR99021 at different time points, the mechanism for mNRPCs’ self-renewal was investigated by transcriptome analysis and western blot assay.

**Results:**

mNRPCs were self-renewing in the presence of bFGF and CHIR99021 and showed prominent RPC characteristics. bFGF was essential in promoting cell cycle by facilitating G1/S and G2/M transitions. bFGF combined with CHIR99021 activated the non-canonical Wnt5A/Ca^2+^ pathway and form a calcium homeostasis. In addition, the self-renewing mNRPCs could differentiate into rod photoreceptor-like cells and retinal pigment epithelium (RPE)-like cells by in vitro induction. When green fluorescent protein (GFP)-labeled cells were transplanted into the subretinal space (SRS) of Pde6b (rd1) mice (also known as RD1 mice, or rodless mice), the cells survived for more than 12 weeks and migrated into the retina. Parts of the recipient retina showed positive expression of photoreceptor marker rhodopsin. Transplanted cells can migrate into the retina, mainly into the inner cell layer (INL) and ganglion cell layer (GCL). Some cells can differentiate into astrocytes and amacrine cells. Cultured mNRPCs did not form tumors after transplanted into NOD/SCID mice for 6 months.

**Conclusions:**

Present study developed an approach to maintain long-term self-renewal of RPCs from adult retinal tissues and revealed that activation of the non-canonical Wnt5A/Ca^2+^ pathway may participate in regulating RPC self-renewal in vitro. This study presents a very promising platform to expand RPCs for future therapeutic application.

**Electronic supplementary material:**

The online version of this article (10.1186/s13287-018-1091-y) contains supplementary material, which is available to authorized users.

## Background

The blindness caused by the eye diseases such as age-related macular degeneration (AMD), retinitis pigmentosa (RP), glaucoma, and Stargardt disease has been associated with retinal ganglion cell (RGC), photoreceptor cell, or retinal pigment epithelium cell (RPE) damage or death. The most popular approach to obtain RPE cells or RGCs is to induce these cells from pluripotent stem cells, including embryonic stem (ES) or induced pluripotent stem (iPS) cells [1–7]. Robert Lanza of Advanced Cell Technology reported two prospective phase 1/2 studies of subretinal transplantation of human embryonic stem cell-derived RPE (hESC-RPE) cells into nine Stargardt’s and nine AMD patients; the results showed safety and graft survival to some extent [8, 9]. However, transplantation of RPE cells can delay retinal degeneration only if surviving photoreceptor cells remain in the eye. Therefore, some researchers tried to use retinal progenitor cells (RPCs) in ocular cell therapy [10–13].

RPCs have been investigated since the beginning of this century. Cells have been isolated from the fetal retina [14–16], postnatal retina [17], and ciliary margin [18–20]. Some researchers have even suggested that Müller cells can be identified as RPCs [21]. However, all of the cells mentioned above cannot be maintained and amplified efficiently in vitro for long periods. It could be important to develop a self-renewing culture of RPCs to generate sufficient cells for therapeutic applications.

Here, we showed that mouse neural retina progenitor cells (mNRPCs) could be established and propagated from adult retina tissue via a chemically defined medium (CDM) including bFGF and CHIR99021. Our study suggested that bFGF was an efficient cytokine that induced mNRPC proliferation by promoting the G1/S and G2/M transitions. The combination of bFGF and CHIR99021 maintained stable mNRPC self-renewal, activated the Wnt5A/Ca^2+^ pathway, and forms a calcium homeostasis in the cells. Cultured mNRPCs express typical RPC markers, including PAX6, SIX3, NESTIN, SOX2 and RCVRN. The cells could express the RPE cell-specific marker RPE-65 in a sandwich-like system and differentiated into rod photoreceptor-like cells in a three-dimensional (3D) culture condition. In particular, after transplantation mNRPCs showed great viability and migration in the recipient retina. It is the first study to maintain the stable self-renewal of RPCs under chemically defined condition, which could provide a renewable source for RPCs and their descendent functional cells for research and transplantation.

## Methods

### Adult mouse neural retinal progenitor cell (mNRPC) isolation and culture

First, neural retinal tissues from 8- to 10-week-old mice were digested with 2% dispase at 37 °C for 20 min. The cells were then treated with 0.25% trypsin for 20 min. The dissociated cells were plated onto 2% Matrigel (Coring, Tewksbury, MA)-coated dishes and cultured in basic medium (BM, DMEM/F12 medium supplemented with 1 × N2, 1 × B27, 0.11 mM beta-mercaptoethanol) containing 10 ng/ml bFGF and 2 μM CHIR99021 (Selleck Chemicals, Houston, TX). All tissue culture products were obtained from Thermo Fisher Scientific except where mentioned.

### Reverse transcription-polymerase chain reaction

Total RNAs were extracted using TRIzol reagent and treated with RNase-free DNase I (both from TaKaRa, Dalian, China). Reverse transcription (RT) was performed with 2 μg RNA, random nonamers (TaKaRa), and Moloney murine leukemia virus reverse transcriptase (Promega, Madison, WI) according to the manufacturer’s instructions. Gene-specific primers are listed in Additional file 1: Table S1. The polymerase chain reaction (PCR) conditions were 95 °C for 5 min, 94 °C for 30 s, annealing temperature for 30 s, and 72 °C for 30 s for 30 cycles, followed by 72 °C for 10 min, using Taq PCR Master Mix (Tiangen Biotech Co., Ltd., Beijing, China).

### Immunofluorescence assay

Cells were fixed in 4% paraformaldehyde (PFA, Sigma-Aldrich, St. Louis, MO), washed three times with phosphate-buffered saline (PBS) containing 0.1% Triton X-100 (Sigma-Aldrich), and incubated in blocking buffer (0.1% Triton X-100) and 5% bovine serum albumin (BSA, Sigma-Aldrich) in PBS for 1 h at room temperature. The cells were then incubated with primary antibodies in blocking buffer overnight at 4 °C, then stained with compatible Alexa 488- or Alexa 555-conjugated secondary antibodies (Thermo Fisher Scientific) in PBS for 30 min at room temperature. The antibodies are described in Additional file 1: Table S2.

### Flow cytometry

Cells were dissociated into a single-cell suspension with StemPro Accutase (Thermo Fisher Scientific) and stained with primary antibodies labeled with fluoroprobes for 30 min at room temperature at 1 μg of antibody per 1,000,000 cells in 0.1 ml of PBS (without Ca^2+^/Mg^2+^) to label the surface markers. Unstained cells and cells stained with isotype control antibody were used as blank and negative controls. The directly conjugated primary antibodies included phycoerythrin (PE)-conjugated CD15, CD24, CD47, CD73, and CD133 (Prominin-1) (BioLegend, San Diego, CA). The fluorescence-labeled cells were analyzed with an LSRIIflow cytometer (Beckman Coulter, Inc., Brea, CA). Debris and doublets were excluded by forward scatter and side scatter manipulations. Gating was implemented based on isotype control staining profiles. All data were analyzed with FlowJo Software (FlowJo, LLC, Ashland, OR).

### Cell viability assay

Cell viability and proliferation were evaluated with the cell counting kit-8 (CCK8, Yeasen, Shanghai, China) according to the instructions. The cells were seeded at a density of 1 × 10^4^ cells per 100 μl per well in 96-well microtiter plates (Corning) and cultured in BM, BM with bFGF (B), BM with CHIR99021 (C) or BM with both bFGF and CHIR99021 (BC), for 1, 3, 5, and 7 days. Then, the absorbance at 450 nm was measured with a microplate reader (iMark™ Microplate Absorbance Reader, Bio-Rad, Hercules, CA).

### Colony formation assay

The cells were plated at 100 cells per 60-mm dish and cultured in BM overnight before changing into medium with bFGF (B), CHIR99021 (C), or both (BC). After culture for 7 days, the cells were fixed with 4% PFA and stained with 1% methylene blue. The visible colonies were counted. All experiments were performed in triplicate, and the values are presented as the mean ± SD.

### RNA-Seq analysis

RNAs of mNRPCs with different treatments were extracted after 1, 3, 5, and 7 days using TRIzol reagent (Thermo Fisher Scientific, Carlsbad, CA). Sequencing was performed on the BGISEQ-500 platform. The RNA-seq reads were subjected to a quality control analysis and mapped to the *Mus musculus* genome using Bowtie 2 with slightly modified default parameters. Fragments per kilobase of transcript per million mapped reads (FPKM) values were calculated using eXpress, and differential expression analysis was performed by the DESeq (2012) R package software. To obtain the gene expression file of the cells, the fold changes for different treatments at different times relative to the values before treatment were calculated to obtain a fold change difference and were sorted based on values close to 0. All FPKM values were increased with the addition of 1 and were log2 transformed. Principal component analysis (PCA) was performed by the pcaMethods R package software [22]. Gene ontology (GO) and enrichment analyses were based on the DAVID Bioinformatics Resources 6.8 (https://david.ncifcrf.gov/) [23]. The heatmap was obtained by the pheatmap R package.

### Western blot analysis

Cells were cultured with BM, BM with bFGF (B), BM with CHIR99021 (C), and BM with bFGF and CHIR99021 (BC) after 1, 3, 5, and 7 days and were harvested and homogenized in ice-cold RIPA buffer (Sigma-Aldrich) containing 1 ml of protease inhibitor cocktail (Selleck) and 1 ml of phosphatase inhibitor cocktail (Selleck) per 100 ml. Equivalent amounts of 20 μg of protein were heated at 100 °C for 10 min and electrophoresed on 10% SDS-PAGE gels and transferred to PVDF membranes. The membranes were blocked with 5% BSA for 2 h at room temperature before incubation with primary antibodies overnight at 4 °C. Then, Amersham™ ECL™ Western Blotting Detection Reagents were added (GE Healthcare, Pittsburgh, PA) after incubation with horseradish peroxidase-conjugated secondary antibodies (Proteintech, Rosemont, IL) at room temperature for 1 h. The densitometry data were quantified with ImageJ software. The antibodies are described in Additional file 1: Table S2.

### Calcium flux measured by image-based flow cytometry

Cells cultured with or without bFGF and CHIR99021 BM after 7 days were resuspended at 5 × 10^6^ cells per ml in 37 °C PBS (without Ca^2+^/Mg^2+^) with 5 μM Fluo-8 (KeyGen BioTech, Nanjing, China) and incubated at 37 °C for 30 min. The cells were washed with PBS (without Ca^2+^/Mg^2+^) and incubated with Hoechst 33342 (Thermo Fisher Scientific; diluted 1:1000) for 10 min at 37 °C before analysis via image-based flow cytometry. The cells were analyzed by means of the Amnis FlowSight imaging flow cytometry platform (EMD Millipore, Burlington, MA), and the images were analyzed by Amnis IDEAS® image-analysis software (EMD Millipore).

### mNRPC in vitro differentiation

For RPE induction [24–26], the cells mixed with 50 ng/ml fibronectin were cultured on 2% Matrigel-coated cell culture dishes in a Matrigel/fibronectin sandwich culture system for 8 days. Then, the cells were fixed with 4% PFA and analyzed by immunofluorescence assay.

For photoreceptor induction [27, 28], the cells were plated on ultralow attachment dishes (Corning) to generate floating spheres for 3 weeks. The spheres were fixed with 4% PFA, embedded in optimal cutting temperature compound (OCT, Tissue-Tek®, Torrance, CA), cut into 8-μm cryosections, and analyzed by immunofluorescence assay.

### Retroviral infection

The day before transduction, Plat-E cells were seeded at 5 × 10^6^ cells per 100-mm dish. The next day, the pMX-IRES-GFP retroviral vector was transfected into Plat-E cells using Lipofectamine™ 3000 transfection reagent (Thermo Fisher Scientific) according to the manufacturer’s instructions. Ten micrograms of plasmid DNA was diluted with 500 μl of Opti-MEM medium (Thermo Fisher Scientific), 25 μl of P3000™ reagent was added carefully to the diluted DNA solution, and 37.5 μl of Lipofectamine™ 3000 was diluted with 500 μl of Opti-MEM medium. Diluted DNA was added dropwise to the diluted Lipofectamine™ 3000 reagent at a 1:1 ratio and incubated for 10 min at room temperature. After incubation, the DNA-lipid complex was added dropwise to Plat-E cells. The cells were then incubated overnight at 37 °C with 5% CO_2_. After 48 h, the virus-containing supernatant was filtered through a 0.45-μm cellulose acetate filter (EMD Millipore) and supplemented with 5 μg/ml polybrene (Sigma-Aldrich). Target cells were incubated overnight in the virus/polybrene-containing supernatant, and then, the incubation medium was replaced with 10 ml of fresh medium.

### Transplantation of mNRPCs into the SRS of RD1 mice

Cells labeled with GFP were digested into single-cell suspensions and unilaterally transplanted into the SRS of 2-week-old RD1 mice (*n* = 6). The mice were anesthetized with 2% sodium pentobarbital. A 33-gauge needle was inserted into the SRS of the central retina for transplantation after a 30-gauge needle was inserted into the vitreous chamber behind the limbus to create a channel. The mice transplanted with 5 × 10^4^ GFP-mNRPCs in 3 μl of 0.9% NaCl were euthanized 8 or 12 weeks after transplantation. The contralateral eyes received a sham injection of 0.9% NaCl alone or without treatment as controls. The eyes were removed and fixed with 4% PFA overnight at 4 °C and embedded in OCT compound. Frozen eyes were cut into 10-μm cryosections for immunohistochemical and terminal deoxynucleotidyl transferase dUTP nick end labeling (TUNEL) assays using in situ cell death detection kit-TMR Red (Roche Diagnostics, Mannheim, Germany) to analyze the survival, and differentiation of GFP-mNRPCs in the recipient retina. To determine whether mNRPCs were tumorigenic in vivo, the cells were transplanted via subcutaneous injection to the nonobese diabetic/severe combined immunodeficiency (NOD/SCID) mice (*n* = 6). All animal procedures were performed according to institutional guidelines and the Guide for the Care and Use of Laboratory Animals issued by the NIH and the guidelines of the animal experimentation ethics committee of Tongji University and in accordance with the Association for Research in Vision and Ophthalmology Statement for the use of Animals in Ophthalmic and Vision Research.

### Statistical analyses

Statistical analysis was performed with GraphPad Prism6 (Graphpad Software, Inc., La Jolla, USA). Colony formation assay and Ki67 positive cell quantification statistical analyses were performed using one-way ANOVA and Sidak’s multiple comparisons test for independent samples. *P* values < 0.05 were considered statistically significant and abbreviated as *P < 0.05, **P < 0.01, and ***P < 0.001.

## Results

### bFGF and CHIR99021 maintained mNRPC self-renewal

**Fig. 1:**
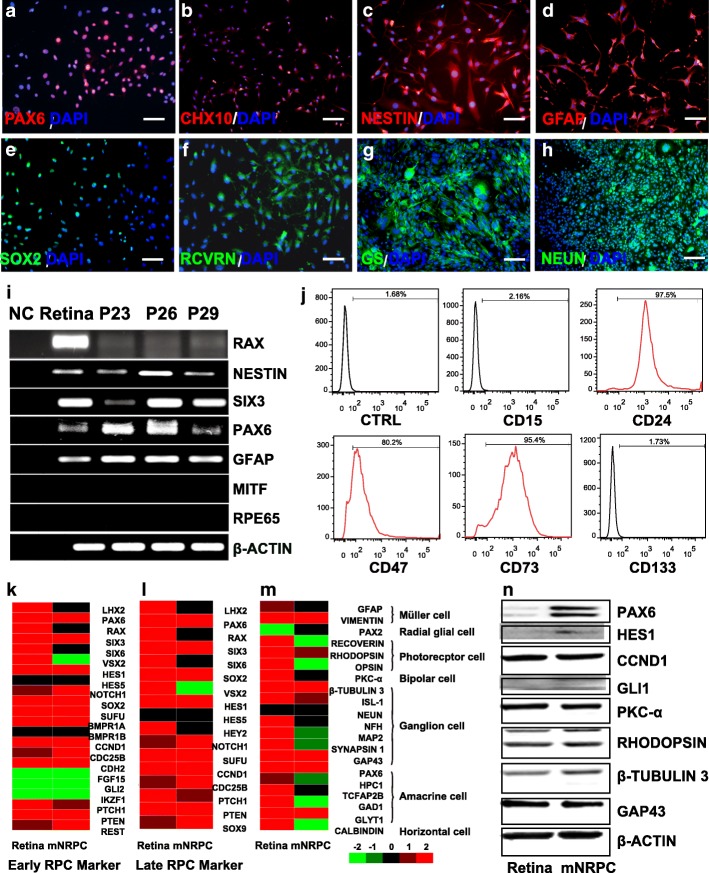
mNRPCs demonstrated typical characteristics of RPCs. **a**–**h** Immunofluorescence assays showed that mNRPCs expressed PAX6 (**a**), CHX10 (**b**), NESTIN (**c**), GFAP (**d**), SOX2 (**e**), RCVRN (**f**), GS (**g**), and NEUN (**h**). Magnification, × 200, scale bar, 100 μm. **i** RT-PCR results showed mNRPCs expressed RAX, NESTIN, SIX3, PAX6, and GFAP, but were negative for MITF and RPE65 at P23, P26, and P29 by mNRPCs. **j** Representative flow cytometry plots showed the expression of CD24 (97.5%), CD47 (80.2%), and CD73 (95.4%), while negative for CD15 and CD133. **k**–**m** Heatmap of the RNA-seq data showed the retinal cell-type specific gene expression profiles of mNRPCs. Black indicates no significant change in expression. Shades of red indicate a significant increase (*P* < 0.05) and green indicates a significant decrease (*P* < 0.05) in mNRPC expression. **n** Western blot confirmed the expression of PAX6, HES1, CCND1, GLI1, PKC-α, RHODOPSIN, β-TUBULIN3, and GAP43. The results are representative of at least three independent experiments, and representative blots are shown

Adult mouse retinal tissue was digested, and the cell suspension was plated onto 2% Matrigel-coated cell culture dishes. The cells were cultured using N2/B27 medium supplemented with well-defined neural progenitor cell mitogens bFGF and EGF, and the small molecular compounds CHIR99021 and SB431542 that had been used to maintain self-renewal of neural stem cells [29, 30]. There were small epithelial-like colonies in the dishes after the first 6 days (Additional file 2: Figure S1a). Once the cells reached 80% confluence, the cells were propagated with Accutase (Additional file 2: Figure S1b-f). By subtracting individual growth factor/small molecule from medium, we found that the combination of bFGF and CHIR99021 was sufficient for mNRPC self-renewal. The cells were cultured using N2/B27 medium with bFGF and CHIR99021 in vitro for up to 30 passages, and the cultured cells expressed retinal progenitor markers including PAX6, SIX3, NESTIN, SOX2, and RCVRN (Fig. 1a–i). For further confirmation of the progenitor characteristics, flow cytometry analysis was performed. The results showed that the mNRPCs expressed RPC surface markers, including CD24, CD47, and CD73 [31, 32], but did not express the photoreceptor precursor marker CD15 [33] and the limbus precursor marker CD133 (Prominin-1) [34] (Fig. 1j). Subsequently, we performed RNA-seq experiments to thoroughly study the biological characteristics of mNRPCs. The data were analyzed by LifeMap Discovery to evaluate early or late RPC markers and seven types of mature retinal cell markers. The results showed that the mNRPCs expressed both early RPC markers (Fig. 1k) and late RPC markers (Fig. 1l). However, the expression of mature retinal cell genes in mNRPCs was generally lower (Fig. 1m). The presence of typical RPC markers, such as PAX6, was confirmed by western blot (Fig. 1n) and Q-PCR (Additional file 3: Figure S2). Embryonic retinas at day 14 and 18 were used as positive control for a comparison of the gene expression profiles in Q-PCR analysis. Gene-specific primers of Q-PCR are listed in Additional file 1: Table S3. In summary, we isolated and established a stable RPC line from adult mouse retinas that can be maintained in vitro for a long time.

### bFGF promoted G1/S and G2/M phase transitions and the combination of bFGF with CHIR99021 activated Wnt5A/Ca^2+^ signaling

**Fig. 2:**
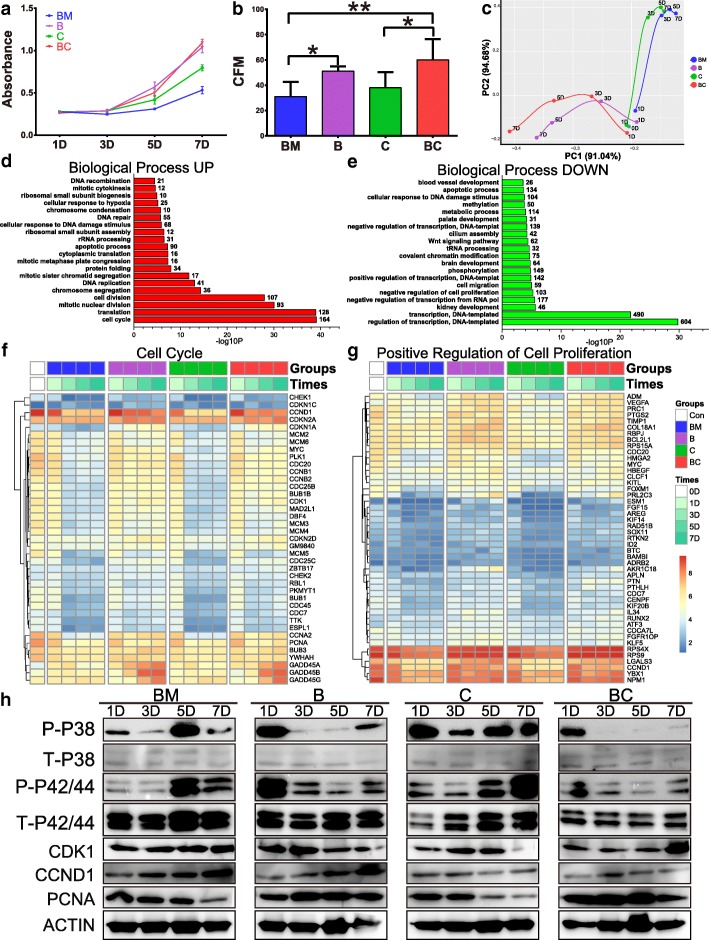
bFGF promoted cell cycle of mNRPCs by stimulating G1/S and G2/M phase transitions. **a** CCK8 assay of the cells with BM, B, C, and BC medium after 1, 3, 5, and 7 days. Data are presented as mean ± SD (*n* = 3). **b** Colony formation assay of the cells with BM, B, C, and BC medium after 7 days. Data are presented as mean ± SD (*n* = 3). *P < 0.05; **P < 0.01 (one-way ANOVA and Sidak’s multiple comparisons test) (GraphPad Prism6). **c** PCA analysis of the mRNA transcriptome in mNRPCs with different treatments at various time. **d**, **e** GO analysis reveals the biological processes altered in mNRPCs on the 7th day. **f**, **g** Heatmap of RNA-seq results showed the expression of genes involved in the biological processes of cell cycle (**f**) and positive regulation of cell proliferation (**g**). **h** Western blot results showed that the MAPK/ERK pathway was activated in all groups and the MAPK/P38 pathway was activated in the BM, B, and C groups but not in BC. The results are representative of at least three independent experiments. Abbreviations: BM, basic medium; B, BM with bFGF; C, BM with CHIR99021; BC, BM with bFGF and CHIR99021; CFM, colony formation

We next analyzed the functions of bFGF (B) and CHIR99021 (C) in mNRPC self-renewal. The cells were cultured with or without bFGF (B) and CHIR99021 (C) in BM and analyzed by the CCK8 assay kit after 1, 3, 5, and 7 days. Both bFGF and CHIR99021 could individually promote the proliferation of the cells compared with BM from day 5 onwards (*P* < 0.001), and the combination of bFGF and CHIR99021 (BC) significantly improved cell proliferation from day 5 onwards compared with the treatment of CHIR99021 (*P* < 0.001) (Fig. 2a). BC did not have a synergistic effect on the cell proliferation compared with B at all test points (*P* > 0.05). However, BC significantly enhanced clonogenicity of mNRPCs. By plating the cells at an ultralow density (100 cells per 60-mm dish), the colony formation ratio was 31%, 51%, 38%, and 60% after 7 days by the treatment with BM, B, C, and BC, respectively (Fig. 2b).

**Fig. 3:**
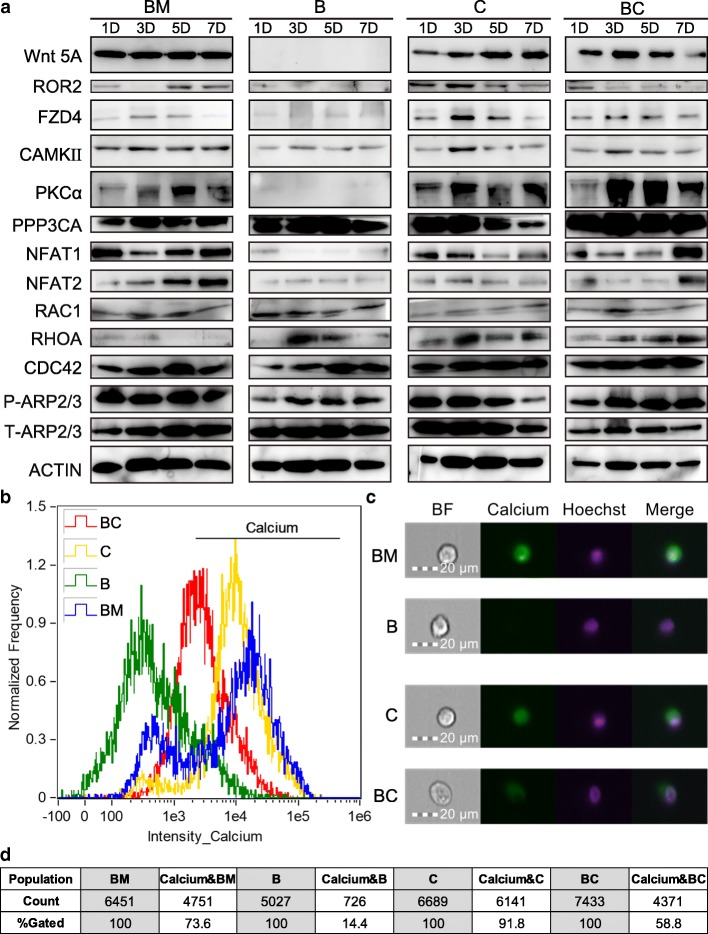
The combination of bFGF with CHIR99021 activated non-canonical Wnt5A/Ca^2+^ signaling pathway and maintained cellular calcium homeostasis. **a** The signature gene expression of Wnt5A/Ca^2+^ signaling pathway was analyzed by western blots. The results are representative of at least three independent experiments and representative blots are shown. **b**–**d** The calcium ion distribution in the cells with different treatments were analyzed on the 7th day using image-based flow cytometry

We next evaluated the transcriptome expression pattern of the cells by above treatment after 1, 3, 5, and 7 days. The cells cultured in BM were used as the control and marked as day 0. Principal component analysis (PCA) was applied to cluster types, by the pcaMethods R package software. Two-dimensional covariance matrix of 1800 genes of 17 samples were clearly classified into 2 populations: without bFGF groups (BM and C) and with bFGF groups (B and BC) (Fig. 2c). Gene ontology (GO) analysis of these two populations on the 7th day revealed that the upregulated genes were enriched in the cell cycle, translation, and cell division categories in groups with bFGF (Fig. 2d), while the downregulated genes were enriched in the DNA-templated transcription categories in the groups without bFGF (Fig. 2e). Heatmap of RNA-seq results showed the expression of genes involved in the biological processes of cell cycle (Fig. 2f) and positive regulation of cell proliferation (Fig. 2g). Next, we evaluated the regulatory pathways that participated in the cell proliferation at the protein level by western blotting. The results showed that the MAPK/ERK pathway was activated in all groups to varying degrees (Fig. 2h); the MAPK/P38 pathway was activated in the BM, B, and C groups but not in the BC group; and MAPK/JNK was not activated in all groups (Additional file 4: Figure S3a). In addition, only the B group showed activation of the canonical Wnt pathway and β-catenin phosphorylation at the S33/37/T41, S675, and S552 sites (Additional file 4: Figure S3a). Given that only the BC could maintain long-term self-renewal of mNRPCs and no activation of the canonical Wnt pathway in BC group, we reasoned that additional pathways may function in mNRPC self-renewal. Based on the expression of ligand, receptors, and the downstream signature genes, we identified Wnt5A/Ca^2+^ pathway as a candidate activated in BC group (Fig. 3a, Additional file 4: Figure S3a). Next, we used FlowSight to measure the calcium flux in the cells on the 7th day. We found that the cells had very high calcium flux in the BM and C groups (73.6% and 91.8%; Fig. 3b–d) and very low calcium flux in the B group (14.4%, Fig. 3b–d). Importantly, the calcium flux in the BC group was approximately 58.8% (Fig. 3 b-d). These results suggested that the activation of Wnt5A/Ca^2+^ pathway in the BC group can form a calcium homeostasis, which may regulate mNRPC self-renewal and need further studies in the future.

### mNRPCs can differentiate into mature retinal cells in vitro

**Fig. 4:**
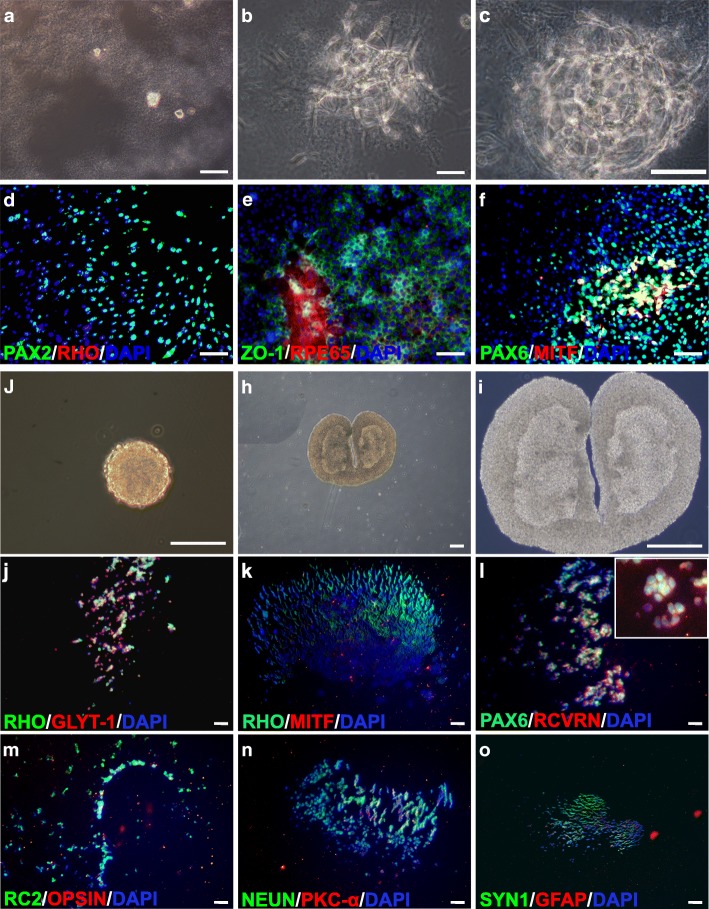
mNRPCs can differentiate into mature retinal cells in vitro*.* **a**–**c** Morphology of mNRPCs in the sandwich induction system on the 1st (**a**), 3rd (**b**), and 5th (**c**) day. **d**–**f** Eight days after sandwich induction, the cells expressed RPE associated genes, including PAX2 (**d**), ZO-1 and RPE-65 (**e**), and PAX6 and MITF (**f**). **g**–**i** Cells cultured in 3D condition will form spheres and can differentiate into rod photoreceptor-like cells. Spheres under bright field after 10 days (**g**) and 3 weeks (**h**, **i**). **j**–**o** Three weeks after 3D induction, differentiated cells expressed GLYT-1 (**j**), RHO (**j**, **k**), PAX6 and RCVRN (**i**), RC2 (**m**), NEUN and PKC-α (**n**), SYN1 (**o**), but did not express MITF (**k**), OPSIN (**m**), and GFAP (**o**). Magnification, × 200 (**a**, **b**, **d**–**f**), × 400 (**c**, **g**, **i**), × 100 (**h**, **j**–**o**); scale bars, 100 μm (**a**–**h**, **j**–**o**), 50 μm (**g**, **i**)

To differentiate mNRPCs into RPE, we used an established sandwich induction condition. The cells were suspended with 50 ng/ml fibronectin and plated onto 2% Matrigel-coated cell culture dishes. The cells proliferated (Fig. 4a-c) and eventually differentiated in the sandwich culture. After 8 days, the cells expressed PAX2 (Fig. 4d), RPE cell-specific markers RPE-65 and ZO-1 (Fig. 4e), PAX6 and MITF (Fig. 4f), but not RHO (Fig. 4d). These results suggest that mNRPCs can differentiate into RPE-like cells under a sandwich culture.

To induce rod photoreceptors, the cells were cultured on the low-attachment culture dishes to form spheres (Fig. 4g–i). Three weeks later, the rod photoreceptor cell-specific markers RHO (Fig. 4j, k) and RCVRN (Fig. 4l) were detected, which indicated the cells differentiate into rod photoreceptor-like cells in a 3D culture condition. Meanwhile, the cells expressed some other mature retinal cell markers, such as the amacrine cell-related markers GLYT-1 (Fig. 4j) and PAX6 (Fig. 4l), the radial glial cell-related marker RC2 (Fig. 4m), the ganglion cell-related markers NEUN (Fig. 4n) and SYN1 (Fig. 4o), and the bipolar cell-related marker PKC-α (Fig. 4n). Above data further confirmed that mNRPCs are multipotent RPCs.

### Transplantation of mNRPCs in RD1 mice

**Fig. 5:**
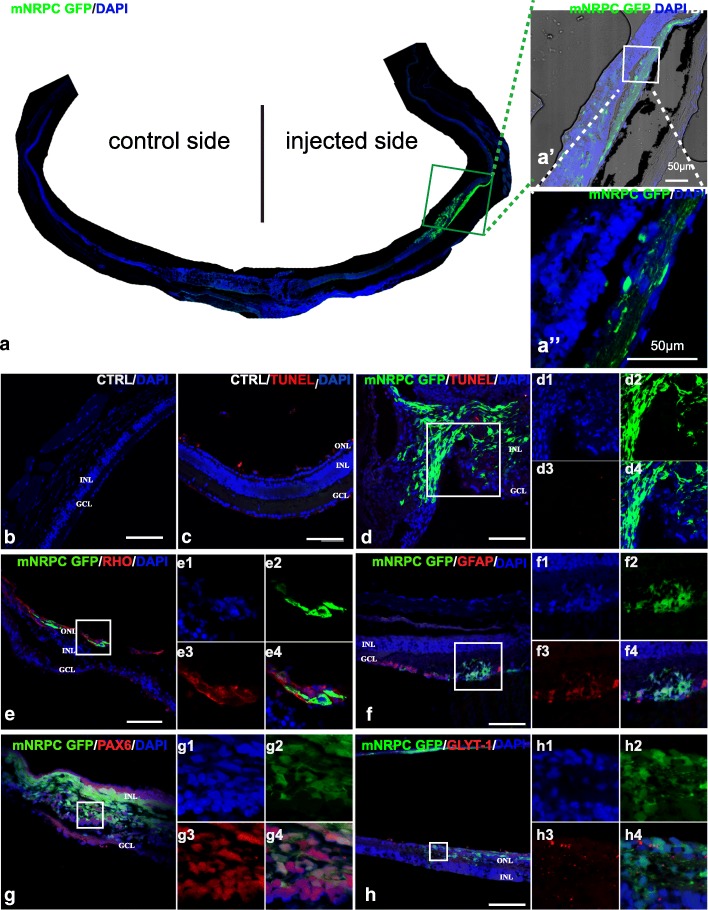
In vivo differentiation of GFP-labeled mNRPCs 12 weeks after transplantation into the SRS of RD1 mice. **(a)** Location of transplanted cells in the recipient retina tissue after 12-week transplantation. A composite image of one retinal cryosection under a × 40 objective lens showing the location of GFP positive transplanted cells in the recipient retina (**a**), highlighted image with phase contrast (**a′**), and highlighted merged image of **a′** (**a″**). The cells were transplanted on the right side. **b** The age-matched RD1 mice retina without cell transplantation. **c**, **d** The TUNEL staining results showed that some GFP-positive cells still exist in the recipient retina after 12-week transplantation (**d**, **d1**–**d4**) compared with 4-week old RD1 mice retina without treatment (**c**). **e**–**h** Representative immunohistochemical staining results of the recipient retina after 12-week transplantation. In recipient retina, positive expression of rhodopsin (red) can be detected (**e**, highlighted in **e1–4**). GFP-positive cells also coexpressed GFAP (red) **(f**, highlighted in **f1–4**), PAX6 (red) (**g**, highlighted in **g1–4**) and GLYT-1 (red) (**h**, highlighted in **h1–4**). Magnification, × 200 (**b**), × 400 (**a**′, **a**″, **c**–**i**); scale bars, 50 μm

In order to evaluate their in vivo potential, mNRPCs labeled with GFP at passages 15–18 were transplanted into the SRS of RD1 mice, in which rod photoreceptor cells completely degenerated by 35 days after birth. The transplanted cells showed obvious migration in the recipient retina after 12 weeks (Fig. 5a, a′–a″). The recipient retina with transplanted cells was obviously thicker than the retina without transplanted cells (Fig. 5a) and control retina of age-matched RD1 mice without transplantation cells (Fig. 5b), indicating certain migration of the transplanted cells. TUNEL results showed that the cells can survive in vivo after 12 weeks (Fig. 5c, d, d1–4). Meanwhile, photoreceptor cell marker rhodopsin can be detected in some parts of retina 12 weeks after transplantation (Fig. 5e), and a few of GFP-positive cells co-expressed rhodopsin (Fig. 5e1–4). Additionally, some GFP-positive cells also expressed GFAP (Fig. 5f, f1–4), PAX6 (Fig. 5g, g1–4), and GLTY-1 (Fig. 5h, h1–4), indicating that transplanted cells can also differentiate into astrocytes and amacrine cells in vivo. The cells did not form tumors when transplanted into nonobese diabetic/severe combined immunodeficiency (NOD/SCID) mice after 6 months, showing the safety of the transplantation in vivo.

## Discussion

Self-renewing RPCs would be essential to generate sufficient progenitors and their descendent functional cells for therapeutic applications. In order to maintain RPCs in vitro, we tried to culture freshly isolated RPCs in a chemically defined medium with different growth factors and chemical compounds (including EGF, bFGF, CHIR99021, and SB431542) that have been reported in neural stem cell maintenance [21, 35–46]. After many trials, we found that the combination of bFGF and CHIR99021 could sustain the long-term in vitro culture of RPCs. We found bFGF and CHIR99021 could activate Wnt5A/Ca^2+^ pathway and maintain cellular calcium homeostasis. In addition, present study showed that self-renewing mNRPCs had the potential to generate photoreceptor-like cells and RPE in vitro. Most importantly, mNRPCs were able to migrate into the INL and GCL and demonstrated multipotent differentiation.

There is still no highly efficient treatment for many genetic or chronic eye diseases that would lead to blindness. Some laboratories reported that transplanting freshly isolated sheets of fetal RPCs with RPE cells at the same time was successful in both animals and humans [47]; however, this approach needs fresh tissues. Tissue shortage and allograft immunogenicity limit the application of this approach. Generation of RPCs from iPSCs was alternative approach and would be especially useful, as it could make the patient tailored cell therapy possible. Our study will be useful to capture and maintain human RPCs from tissues or during iPSC differentiation. Renewable human RPCs could not only provide sufficient progenitors, but also their descendant functional cells for cell therapy for retina-associated diseases.

## Conclusion

In this report, we isolated and maintained adult mouse retinal progenitor cells (RPCs) under a chemically defined culture. The self-renewing mNRPCs could differentiate into rod photoreceptor-like cells and retinal pigment epithelium (RPE)-like cells by in vitro induction. The cells survived for more than 12 weeks, migrated into the retina, and demonstrated multipotent differentiation when transplanted into the SRS of RD1 mouse. Our data revealed that activation of non-canonical Wnt5A/Ca^2+^ pathway may participate in regulating RPC self-renewal in vitro. The study presents a very promising platform to expand RPCs for future therapeutic application.

## Additional files

Additional file 1: **Table S1.** Primers used in RT-PCR experiments. Table S2 Antibodies used in immunostaining, flow cytometry, and WB. Table S3 Primers used in Q-PCR experiments. (DOCX 33 kb)

Additional file 2: **Figure S1.** Morphology of mNRPCs at different passages. (a) Six days of primary culture, (b) 12 days of primary culture, (c) P1, (d) P4, (e) P9, and (f) P27. Magnification, × 100; Scale bar, 200 μm. (PDF 2849 kb)

Additional file 3: **Figure S2.** Q-PCR verification analysis of the gene expression profiles of mNRPCs. Embryonic retinas at 14th and 18th day, and 8-week-old adult mouse retinas used as control. The heatmap shows processed ΔCt values. GAPDH was used as reference gene for normalization. Shades of red indicate a higher ΔCt, and black indicates a lower ΔCt. (PDF 855 kb)

Additional file 4: **Figure S3.** bFGF can activate the canonical Wnt pathway. (a) The western blot results showed that bFGF can activate the canonical Wnt pathways and caused β-catenin phosphorylation at the S33/37/T41, S675, and S552 sites. The results are representative of at least three independent experiments, and representative blots are shown. (b) The co-immunostaining of NFAT1 and KI67 on the 7th day of the cells with BM, B, C, and BC medium. Magnification, × 200; Scale bar, 50 μm. (c) KI67 positive cells were quantified on the 7th day with BM, B, C, and BC medium. The results showed KI67 positive ratio in BM (17.83 ± 0.32%), B (58.08 ± 2.81%), C (19.96 ± 2.35%), and BC (64.19 ± 5.27%). Data are presented as mean ± SD (*n* = 3). ***P < 0.001 (one-way ANOVA and Sidak’s multiple comparisons test). (PDF 11714 kb)

## Abbreviations

3D: Three-dimensional
AMD: Age-related macular degeneration
BM: Basic medium
BSA: Bovine serum albumin
CDM: Chemically defined medium
CFA: Colony formation assay
GCL: Ganglion cell layer
GFP: Green fluorescent protein
GO: Gene ontology
ICC: Immunocytochemistry
INL: Inner cell layer
iPS: Induced pluripotent stem
mNRPCs: Mouse neural retina progenitor cells
NOD/SCID: Nonobese diabetic/severe combined immunodeficiency
PBS: Phosphate-buffered saline
PCA: Principal component analysis
PCR: Polymerase chain reaction
PFA: Paraformaldehyde
RGC: Retinal ganglion cell
RP: Retinitis pigmentosa
RPC: Retinal progenitor cell
RPE: Retinal pigment epithelium
SRS: Subretinal space
TUNEL: Terminal deoxynucleotidyl transferase dUTP nick end labeling
