# Supplementary material for: The combination of bFGF and CHIR99021 maintains stable self-renewal of mouse adult retinal progenitor cells
Source: Stem Cell Res Ther. 2018 Dec 13;9:346. doi: 10.1186/s13287-018-1091-y (PMC6292077; doi:10.1186/s13287-018-1091-y)
Supplement: Supplementary file 1 — Table S1. Primers used in RT-PCR experiments. Table S2 Antibodies used in immunostaining, flow cytometry, and WB. Table S3 Primers used in Q-PCR experiments. (DOCX 33 kb) [file 13287_2018_1091_MOESM1_ESM.docx]

**Supplemental** **Methods**

**Quantitative Real-Time PCR (Q-PCR)**

Total RNA was extracted and [reverse transcription](https://www.sciencedirect.com/topics/neuroscience/reverse-transcriptase) was performed using the Primescript™ RT Master Mix kit (Takara, Japan). Real-time [PCR](https://www.sciencedirect.com/topics/medicine-and-dentistry/polymerase-chain-reaction) was performed in a CFX Connect™ Real-Time PCR Detection System (Bio-Rad, Hercules, USA) by using SuperReal Premix plus (SYBR green) kit (Tiangen Biotech, Beijing, China). PCR amplification was carried out in triplicate and the conditions were 95 °C for 5 min, 94°C for 30 sec, 60 °C for 40 s for 40 cycles. A melting curve was generated to analyze the specificity of the reactions. The relative expression level was calculated as ΔCt [ΔCt = Ct^Target^ − Ct^GAPDH^]. The heatmap of ΔCt was obtained by the Heml software. [Primer](https://www.sciencedirect.com/topics/medicine-and-dentistry/primer-molecular-biology) sequences are listed in [Table 2](https://www.sciencedirect.com/science/article/pii/S0014483518301647?via%3Dihub#tbl1).

**Table S1: Primers used in RT-PCR experiments**

| **Genes** | **Forward (5’-3’)** | **Reverse (5’-3’)** |
| --- | --- | --- |
| RAX | CCCTGAGGCTAAACTTGCAG | GTTCCCTTCTCCTCCTCCAC |
| NESTIN | TCAGCGTCAAGAGGAAGTGAG | TGATAGGGACAGGGGTAGGG |
| SIX3 | CGACTCGGAATGTGATGTATG | TGGAGTAGAAGAAGAGGAAGAG |
| PAX6 | GCAATGGCCTCCTCCATGAT | AGTGATGTCGTTGTTGGCCT |
| GFAP | ATACCCTTCACCTTCAACTAAC | CTCTGCTCATCTTTCCTCTTC |
| MITF | TGTGACCTCCTTTGAAATTAAC | CCTCCTCTTCTCCTTCTTCT |
| RPE-65 | ACTAACAGCTCATGTCACAGGC | AAACAGGTGATAGAAAGGCTCAG |
| β-ACTIN | TGACGTTGACATCCGTAAAG | AGGAGCCAGAGCAGTAAT |

RAX: retina and anterior neural fold homeobox; SIX3: sine oculis-related homeobox 3; PAX6: paired box 6; GFAP: glial fibrillary acidic protein; MITF: microphthalmia-associated transcription factor; RPE-65: retinal pigment epithelium 65.

**Table S2: Antibodies used in immunostaining，Flow Cytometry and WB**

| **Antibodies** |  | **Source** | **Dilution** |
| --- | --- | --- | --- |

| **Immunostaining** |  | |  |
| --- | --- | --- | --- |
| PAX6 | Covance, PRB-278P | 200 | |
| SOX2 | Millipore, AB5603 | 500 | |
| CHX10 | Abcam, ab16141 | 500 | |
| SIX3 | Novus Biologicals, NBP2-21662 | 200 | |
| NESTIN | Millipore, MAB353 | 200 | |
| MITF | Abcam, ab13703 | 200 | |
| VIMENTIN | Millipore, AB1620 | 200 | |
| OPSIN | Millipore, AB5405 | 200 | |
| RHODOPSIN | Millipore, MAB5316 | 200 | |
| PKC-α | Sigma, P4334 | 500 | |
| NEUN | Millipore, MAB377 | 200 | |
| RCVRN | Millipore, AB5585 | 200 | |
| GS | Sigma, G2781 | 500 | |
| ZO-1 | Invitrogen, 61-7300 | 500 | |
| GFAP | Sigma, SAB4501162 | 500 | |
| Alexa conjugated secondary antibodies | Thermo Fisher Scientific | 1000 | |
| **Flow Cytometry** |  |  | |
| CD15 | BioLengend, 125605 | 1000 | |
| CD24 | BioLengend, 138503 | 1000 | |
| CD47 | BioLengend,127507 | 1000 | |
| CD73 | BioLengend,127205 | 1000 | |
| CD133 | BioLengend,141203 | 1000 | |
| **WB** |  |  | |
| PAX6 | Covance,PRB-278P | 500 | |
| HES1 | Abcam,ab49170 | 1000 | |
| RAC1 | Abcam,ab155938 | 1000 | |
| CDC42 | Abcam,ab64533 | 1000 | |
| RhoA | Abcam,ab54835 | 1000 | |
| GAP43 | Millipore,MAB347 | 1000 | |
| RHO | Millipore,MAB5316 | 500 | |
| ARP2/3 | Millipore,ABN176 | 1000 | |
| β-Ⅲ-TUBULIN | Promega,G7121 | 1000 | |
| Anti-p34-Arc/ARPC2 Antibody | Upstate,07-227 | 1000 | |
| PKC-α | Sigma,P4334 | 1000 | |
| CCND1 | Proteintech, 60186-1-Ig | 1000 | |
| CALCINEURIN | Proteintech, 13422-1-AP | 1000 | |
| FZD2 | Proteintech, 24272-1-AP | 500 | |
| FZD4 | Proteintech, 15328-1-AP | 500 | |
| FZD8 | Proteintech, 55093-1-AP | 500 | |
| AXIN2 | Proteintech, 20540-1-AP | 1000 | |
| JNK | Proteintech, 51151-1-AP | 1000 | |
| CDK1 | Proteintech, 19532-1-AP | 1000 | |
| CAMK2 | Proteintech, 13730-1-AP | 1000 | |
| β-Catenin | CST,#8480 | 1000 | |
| WNT5A/B | CST,#2530 | 1000 | |
| WNT5A | CST,#2392 | 1000 | |
| (Actived) β-Catenin | CST,#8814 | 1000 | |
| P-β-Catenin (Ser675) | CST,#4146 | 1000 | |
| P-β-Catenin (Ser33/37/Thr41) | CST,#9561 | 1000 | |
| P-β-Catenin (Ser552) | CST,#9566 | 1000 | |
| GSK-3β | CST,#9315 | 1000 | |
| P-GSK3β(Ser9) | CST,#5558 | 1000 | |
| p44/42 MAPK | CST,#4695 | 1000 | |
| P-p44/42 MAPK (Thr202/Tyr204) | CST,#4370 | 1000 | |
| p38 MAPK | CST,#8690 | 1000 | |
| P-p38 MAPK (Thr180/Tyr182)(D3F9) | CST,#4511 | 1000 | |
| PCNA | CST,#13110 | 1000 | |
| C-MYC | CST,#13987 | 1000 | |
| P-JNK (Thr183/Tyr185) (G9) | CST,#9255 | 1000 | |
| GLI | CST,#2553 | 1000 | |
| NFAT1 | CST,#5861 | 1000 | |
| NFAT2 | CST,#8032 | 1000 | |
| NFAT3 | CST,#2188 | 1000 | |
| ROR2 | CST,#88639 | 500 | |
| FZD6 | CST,#5158 | 500 | |
| LRP6 | CST,#3395 | 500 | |
| FRZB | Invitrogen, PA5-44188 | 500 | |
| HRP-conjugated β-Actin Antibody | Proteintech, HRP-60008 | 2000 | |

PAX6: paired box 6;

SOX2: SRY (sex determining region Y)-box 2;

CHX10: synonyms of VSX2, visual system homeobox 2;

SIX3: sine oculis-related homeobox 3;

MITF: microphthalmia-associated transcription factor;

PKC-α: protein kinase C alpha;

NEUN: neuronal nuclei;

RCVRN: recoverin;

GS: glutamine synthetase;

ZO-1: synonyms of TJP1, tight junction protein 1;

GFAP: glial fibrillary acidic protein;

HES1: hes family bHLH transcription factor 1;

RAC1: Rac family small GTPase 1;

CDC42: cell division cycle 42;

RhoA: ras homolog family member A;

GAP43: growth associated protein 43;

RHO: rhodopsin;

ARP2/3: actin related protein 2/3 complex;

CCND1: cyclin D1;

CALCINEURIN: Old name of PPP3CA, protein phosphatase 3;

FZD: frizzled class receptor;

CDK1: cyclin-dependent kinase1;

CAMK2: calcium/calmodulin-dependent protein kinase II;

CTNNB: β-Catenin;

PCNA: proliferating cell nuclear antigen;

C-MYC : myelocytomatosis oncogene C;

NFAT: nuclear factor of activated T cells;

ROR2: receptor tyrosine kinase-like orphan receptor 2;

LRP6: low-density lipoprotein receptor (LDLR) protein;

FRZB: secreted frizzled-related protein 3.

**Table S3: Primers used in Q-PCR experiments**

| **Genes** | **Forward (5’-3’)** | **Reverse (5’-3’)** |
| --- | --- | --- |

| LHX2 | GCCTCAAGTGCTGTGAAT | CGCCTGTAGTAGTCTTCTTTG |
| --- | --- | --- |
| PAX6 | ACACAGCAGTTGGGTATTC | GTCCTTGGTTCTAGTCCATTC |
| RAX | AAGGCCAAAGAGCACATC | GCAGGGCTAGCAAAGAAA |
| SIX3 | CGACTCGGAATGTGATGTATG | TGGAGTAGAAGAAGAGGAAGAG |
| SIX6 | CGCAGCATGGAGTCTTATT | CGGTTAGTTAGCGGTTTATCT |
| HES1 | GGCGAAGGGCAAGAATAA | GAATGTCTGCCTTCTCTAGC |
| VSX2 | CTCCCAGAAGACAGGATACA | GGCTCCATAGAGACCATACT |
| SOX2 | GGGAGAAAGAAGAGGAGAGA | CGCGATTGTTGTGATTAGTTT |
| NOTCH1 | CAACTGTCCTCTGCCATATAC | CTTCAGACTCCTTGCATACC |
| BMPR1A | GTTCGGCAGGTTGGTAAA | GCTAGCTTCTTCAGTGGTAAA |
| IKZF1 | TCTCAGCTCTCCTGACATATAG | CTAGAATGTTCCTGGCATCTAC |
| CCND1 | GGGACAACTCTTAAGTCTCAC | GGCAGCCTTTCCCATAAA |
| FGF15 | CCAGTCTGTGTCAGATGAAG | GGAAGCAGTTGGAGACATAG |
| PTCH1 | CTCCTAGGTAAGCCTCCTTTA | GCTGTCCACTTGGTAGTTTAT |
| REST | CTGGGAAGTTTGGAGCTAAG | CTGGAGCGGGATCTTTATTG |
| HES5 | TCACCTCAAGGTCCACAT | CCCACCCATACAAAGGAATC |
| SUFU | GAGTCCTTCTGCCAACATC | GTCCGTCTGTTCCTGTAAAC |
| GLI2 | GTTGCTGTGGACTAGGAATAG | GAGAAATAGGAGCGGGTAAAC |
| BMPR1B | GGCTCACAAGTCCCTAATAATC | CATTGCCTTTCCATCCATAAAC |
| CDC25B | TGAGGCCACCTACCTTATAG | AGTGGAGTGAGACAGGAAA |
| CDH2 | TGAAACGGCGGGATAAAG | CCTCCACCTTCTTCATCATATT |
| PTEN | CAGTAGAGGAGCCATCAAATC | GAGTCAGTGGTGTCAGAATATC |
| HEY2 | AAGATGCTCCAGGCTACA | CACTTCTGTCAAGCACTCTC |
| SOX9 | GAAAGGAAGGAAGGAAGGAAG | AAGCTCACCAATGCTCTATG |
| GFAP | ATACCCTTCACCTTCAACTAAC | CTCTGCTCATCTTTCCTCTTC |
| PAX2 | CTAAGATGGCAGAACCAACC | CTACCGAGAGAGCAACTACA |
| RHODOPSIN | CCTGTTGGAAGGCTACTTTAG | AGGAGAAGGCAGGAGTAAA |
| PKC-α | CAAAGAGGAGGAAGCAGAAG | GGAAAGCCCATGCAGATTA |
| ISL-1 | CATCTAGAGGAAGAGCAGAAAC | GCAAGGCAGTGACCAATA |
| NFH | ACAAGAGAAACACCCAGAATAG | ACGGAGGGAAAGGAAGAA |
| SYNAPSIN 1 | CCACTTCTCATTCCTCAGTATG | TGACCAGGAACAGGATTCTA |
| HPC1 | TCAGTCAGACTACCGAGAAC | TCCAGCATGTCTTCCAATTC |
| GAD1 | CTGTAGAGACACCCTGAAGTA | AGCCATTCACCAGCTAAAC |
| CALBINDIN | CTCTGTGTGCTCTCTGATTAAA | GATACCCTTGGTGGAAATGAA |
| VIMENTIN | GAGAAATTGCAGGAGGAGATG | TCAAGACGTGCCAGAGAA |
| RECOVERIN | CTGAGAAGATCTGGGCATTC | CAGTCGCAGAATTTCCTTATTG |
| OPSIN | TATGCCTTCCACCCTCTT | TGCCGGTTCATAAAGACATAG |
| β-TUBULIN 3 | CATTCTGGTGGACTTGGAAC | CACCACTCTGACCAAAGATAAA |
| NEUN | GGCCGTGCTGTGTATAAT | GCTGCATAGCCTCCATAAA |
| MAP2 | CCAGTTTCTCTCTGGCTTTAG | CCCAGAGTGTGTGAGTTTATT |
| GAP43 | GCTGTGCTGTATGAGAAGAA | TTTGGTCGCAGCCTTATG |
| TCFAP2B | GCAACATCTCTGCTCCTATC | CTGCCCTTTCTACTCCTTTC |
| GLYT1 | TCCAGGGTCTCTATCCTTTG | CCCTTCTTTCCCTCCATAAAC |

RAX: retina and anterior neural fold homeobox; SIX3: sine oculis-related homeobox 3; PAX6: paired box 6; GFAP: glial fibrillary acidic protein; MITF: microphthalmia-associated transcription factor;
