# Supplementary figures and images for: The combination of bFGF and CHIR99021 maintains stable self-renewal of mouse adult retinal progenitor cells
Source: Stem Cell Res Ther. 2018 Dec 13;9:346. doi: 10.1186/s13287-018-1091-y (PMC6292077; doi:10.1186/s13287-018-1091-y)

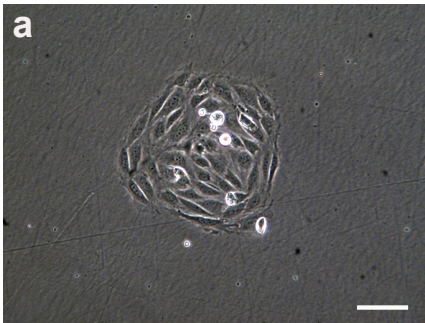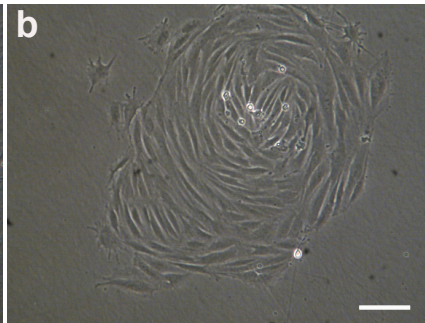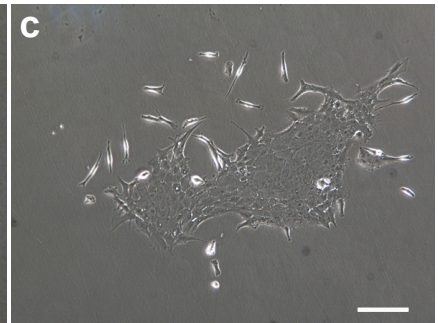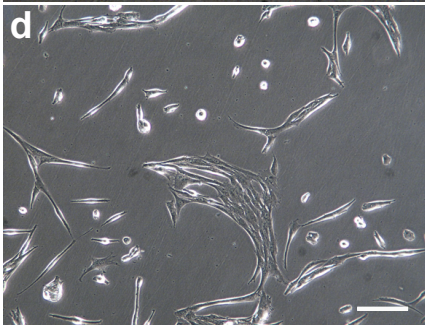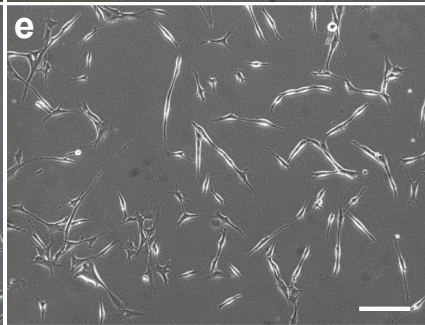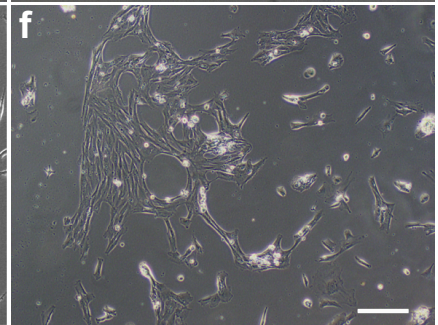

Supplement: Supplementary file 2 — Figure S1. Morphology of mNRPCs at different passages. (a) Six days of primary culture, (b) 12 days of primary culture, (c) P1, (d) P4, (e) P9, and (f) P27. Magnification, × 100; Scale bar, 200 μm. (PDF 2849 kb) [file 13287_2018_1091_MOESM2_ESM.pdf]

**a**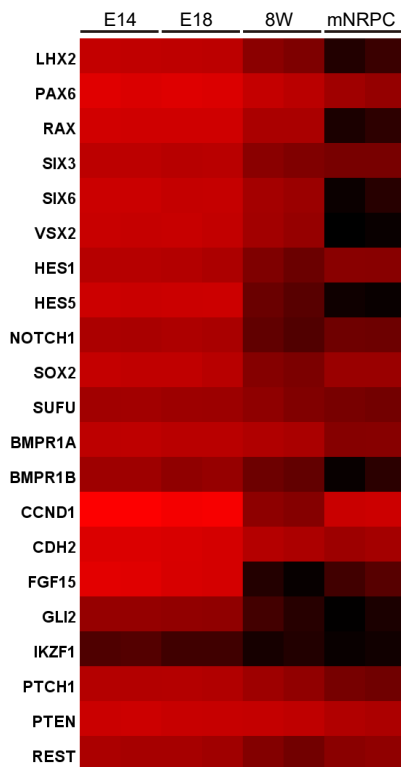

Early RPC Marker

**b**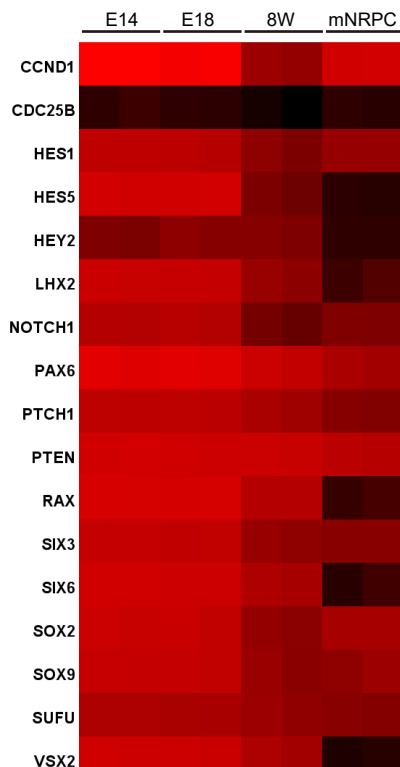

Late RPC Marker

**c**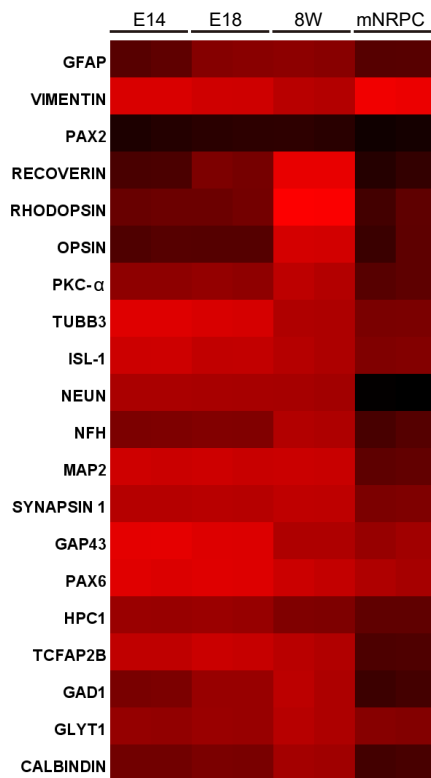

-22 -18 -14 -10 -6 -2

Supplement: Supplementary file 3 — Figure S2. Q-PCR verification analysis of the gene expression profiles of mNRPCs. Embryonic retinas at 14th and 18th day, and 8-week-old adult mouse retinas used as control. The heatmap shows processed ΔCt values. GAPDH was used as reference gene for normalization. Shades of red indicate a higher ΔCt, and black indicates a lower ΔCt. (PDF 855 kb) [file 13287_2018_1091_MOESM3_ESM.pdf]

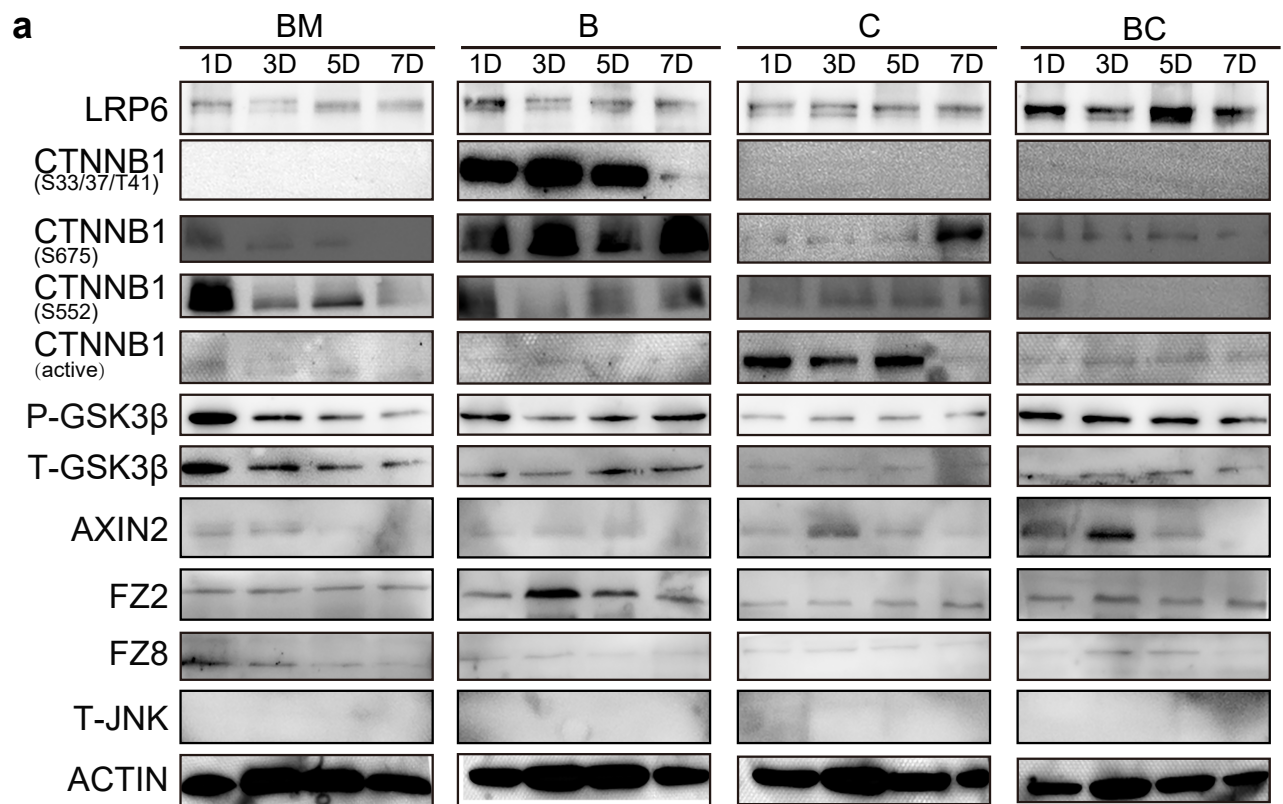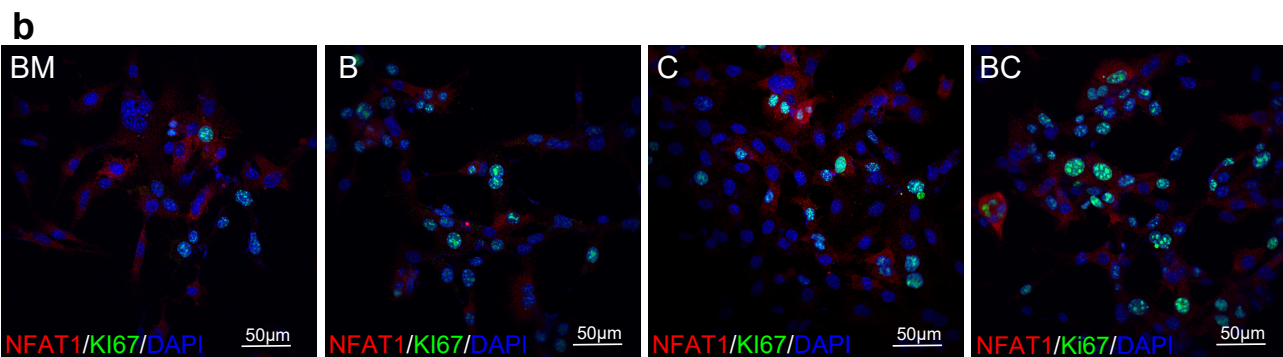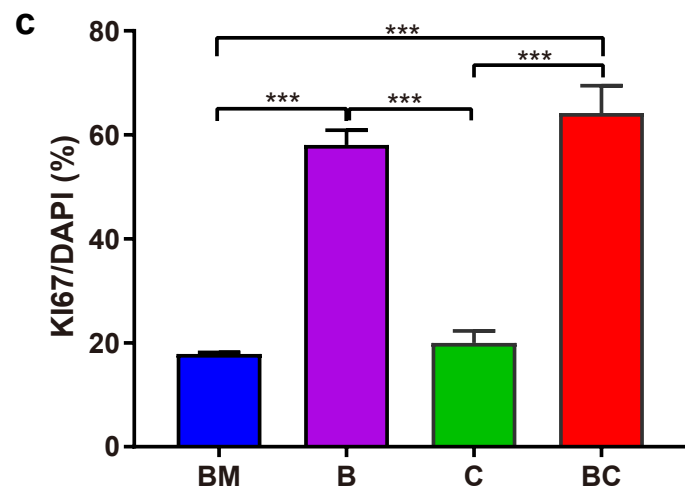

Supplement: Supplementary file 4 — Figure S3. bFGF can activate the canonical Wnt pathway. (a) The western blot results showed that bFGF can activate the canonical Wnt pathways and caused β-catenin phosphorylation at the S33/37/T41, S675, and S552 sites. The results are representative of at least three independent experiments, and representative blots are shown. (b) The co-immunostaining of NFAT1 and KI67 on the 7th day of the cells with BM, B, C, and BC medium. Magnification, × 200; Scale bar, 50 μm. (c) KI67 positive cells were quantified on the 7th day with BM, B, C, and BC medium. The results showed KI67 positive ratio in BM (17.83 ± 0.32%), B (58.08 ± 2.81%), C (19.96 ± 2.35%), and BC (64.19 ± 5.27%). Data are presented as mean ± SD (n = 3). ***P < 0.001 (one-way ANOVA and Sidak’s multiple comparisons test). (PDF 11714 kb) [file 13287_2018_1091_MOESM4_ESM.pdf]
